# More is needed to end TB: commentary on the United Nations High-Level Meeting on TB

**DOI:** 10.5588/ijtldopen.23.0576

**Published:** 2024-01-01

**Authors:** G. B. Migliori, V. Bhatia, L. D'Ambrosio, M. C. B. Raviglione, S. Rijal

**Affiliations:** ^1^ Istituti Clinici Scientifici Maugeri, Istituto di Ricovero e Cura a Carattere Scientifico, Tradate, Italy;; ^2^ Department of Communicable Diseases, WHO South-East Asia Regional Office, New Delhi, India;; ^3^ Public Health Consulting Group, Lugano, Switzerland;; ^4^ Centre for Multidisciplinary Research in Health Science (MACH), University of Milan, Italy

**Keywords:** tuberculosis, End TB, United Nations, High-Level Committees

The United Nations High-Level Meeting (UNHLM) on TB in September 2023 represented a unique opportunity to consolidate results achieved in previous major political events such as the World Health Assembly (WHA) in 2014,^1^ the Ministerial Conference in Moscow in 2017^2^ and the previous UNHLM on TB in 2018.^3^ It was a chance to pursue bolder government commitments to further augment the political visibility of TB in the context of the UN Sustainable Development Goals (SDG), thus increasing the resources available to end TB.^4,5^ Following the meeting, the UN adopted Resolution A/RES/73/3, a new political declaration on TB. It consists of 84 points, which include new targets to be reached in the period 2023–2027 (see Table 1), in alignment with the WHO’s flagship initiative to End TB. Most targets aim at enhancing previous UNHLM targets, with the addition of an entirely new Target 5 (to accelerate a new anti-TB vaccine development).

**Table 1.** New TB targets to be reached in the period 2023–2027, adopted by the 2023 UN General Assembly political declaration on TB. Source: WHO Status update: Reaching the targets in the political declaration of the United Nations General Assembly High-level Meeting on the fight against tuberculosis. Geneva, Switzerland: WHO, 2023.

Objectives	Targets
Universal access to WHO-recommended TB treatment for all	90% of people reached with TB treatment between 2023 and 2027 (End TB Strategy target is ≥90% by 2025)
Universal access to WHO-recommended rapid diagnostic tests for all	100% of people diagnosed with TB were tested initially using a WHO-recommended diagnostic test (End TB Strategy target is ≥90% by 2025)
Universal access to TB preventive treatment for all	90% reached with TB preventive treatment between 2023 and 2027 (End TB Strategy target ≥90% by 2025)
Financial risk protection for vulnerable people with TB (process indicator)	100%: All (eligible) people with TB have access to health and social benefits package to ensure that there is no financial hardship due to TB disease
License a new TB vaccine to accelerate TB incidence decline (process indicator)	>1: Licensing of at least one new TB vaccine within 5 years
Sustained and adequate financing for TB services and TB research and innovation (process indicator)	Reaching US$22 billion annually by 2027; US$5 billion per year for research by 2027

Having held preparatory discussions in the WHO South-East Asia Region (SEAR) during a Ministerial Meeting held in Gandhinagar, India, in August 2023,^6^ we reflect here on the outcomes of the UNHLM. Our first suggestion is the need to establish high-level multisectoral mechanisms (in the form of a high-level committee/commission/council [HLC]), which would directly report to respective head of states. This is a key step towards ending TB and has a potential collateral benefit for ending other communicable diseases. SEAR is a pivotal setting to lead discussion on HLCs. With a quarter of the world’s population, it is host to a disproportionately high burden of TB, with over 45% of estimated global TB incidence and close to 50% of TB deaths in 2022.^7^ This leads to devastating social and financial implications for patients and their households. Within the region, it has been estimated that 30–80% of people with TB and their families face catastrophic costs due to the disease.^7^ It is important to recognise that there has been progress in the TB response, with a decline of 31% and 41% in incidence and mortality respectively, between 2000 and 2021. However, this is far from sufficient to reach the UN SDGs and related End TB targets. Ending TB needs sustained political commitment, collaboration among multiple sectors (including those working beyond health), engagement with all partners, including communities, and increased resources. This necessitates the establishment of HLCs.

## HLCs and their role

The concept of establishing HLCs that report directly to the highest political authority (i.e., the head of state, president or prime minister), is not entirely new. It is based on previous recommendations emerging from global events,^2,3^ and can accelerate country action and promote effective multisectoral accountability frameworks (MAF).^8^ Since 2017, establishing high-level multisectoral mechanisms has been part of all SEAR political commitments on TB.^9,10^ It is envisaged that HLCs would have the power to regularly review progress by the various sectors related directly or indirectly to health and TB, and make timely and bold decisions to advance and accelerate the response. An HLC convened by the head of state, alongside a well-organised MAF, are key to such a response, ensuring a link between executive power and faster action. The process of designing and implementing HLCs, and involving diverse sectors and stakeholders, will contribute to ending TB by involving forces outside of national TB programmes or the ministry of health, to foster critical actions. These include the use of a primary healthcare approach and engaging those responsible for addressing social determinants (such as poverty alleviation or food security and related undernutrition), which are major drivers of TB and other conditions. It also facilitates a commitment to rapid uptake of innovation from research and development, ensuring universal access for all in need. Finally, it allows for sustainable financing to end TB and the balancing of international and domestic resources, and the shift from external support in countries experiencing rapid economic development.

## Examples of high-level engagement in TB

In 2019, after a request by the Prime Minister of Vietnam, a National Commission on Ending Tuberculosis was established as ‘an interdisciplinary coordination organization with the task of helping the Prime Minister direct, urge, coordinate and guide ministries and agencies on TB prevention and control to end TB by 2030, contributing to the protection, care and improvement of people's health’. The Vietnam HLC was chaired by the deputy Prime Minister with the Minister of Health as vice-chair.^11,12^ As a result – and despite imitations imposed by the COVID-19 pandemic – Vietnam was able to consolidate its slow downward trends in incidence and mortality due to TB. Other examples of heads of state showing leadership in the fight against TB are those from India, Indonesia and Timor-Leste, where their direct engagement has led to innovative approaches.

## The HIV/AIDS experience

There are positive examples shown in Table 2. UNAIDS describes the crucial role played by the International Partnership against AIDS in Africa (IPAA) launched by Kofi Annan in 2000.^13^ This followed key political events and inclusive consultations engaging governments, the UN, the private sector and civil society. This regional strategy ‘helped to lay the foundations for the global strategies debated and, to a considerable extent agreed,’ at the 2001 United Nations General Assembly Special Session. The results were concrete, high-powered AIDS committees and commissions were established in eSwatini, Ghana, Malawi, Mozambique, Nigeria and Zimbabwe, and the ‘President of Burkina Faso created a National Solidarity Fund through debt relief initiative mechanisms’.^13^

**Table 2.** Examples of HLCs in the HIV/AIDS domain.

Country	Experience
Ethiopia	The Federal HIV/AIDS Prevention and Control Office (HAPCO) was established in 2002 as an agency of the Federal Ministry of Health and the executive arm of the National AIDS Council (NAC), which is chaired by the President
Ghana	Under the 95-95-95 programme, Ghana accomplished a 71% awareness rate among people living with HIV about their HIV status, ensured that 99% of those diagnosed with HIV received consistent ART, and achieved a viral suppression rate of 79% among individuals receiving ART. The impact of the Ghana AIDS Commission (GAC) was quantitatively evaluated in a study. Based on statistically measurable reduction of the number of new cases of HIV infection in the southern part of the country between 2002 and 2027 (15% among females and 18% among males), the Authors concluded that ‘GAC outcomes were a success’
Malawi	In 1998, a Cabinet Committee on AIDS was led by Vice-President Justin Malewezi, a powerful AIDS advocate, comprising all ministers with the mandate to build a broad-based, multisectoral, national response to the disease. From 1998 onwards, AIDS was increasingly mentioned in public statements of the President, Ministers and Members of Parliament. As a result of this bold political move, the 90-90-90 targets were reached in 2020
Rwanda	The Head of State and the First Lady, through their leadership, promoted the reorganisation and restructured coordination of AIDS control activities by creating the National AIDS Control Commission (NACC), which was implemented in November 2000. The NACC works directly under the Office of the President to formulate policies and ensure coordination at the national level, while TRAC, which is a specialised technical unit, is called to support the Ministry of Health (MoH) and NACC in the coordination of medical interventions. Rwanda reached the 90-90-90 targets by 2020
South Africa	The South African National AIDS Council (SANAC) is chaired by the Deputy President of South Africa
Tanzania	The Prime Minister’s Office, through the Tanzania Commission for AIDS (TACAIDS), has developed the 5^th^ National Multisectoral Strategic Framework for HIV 2021–2026
Thailand	The National AIDS Prevention and Control Committee (1990) was chaired by the Prime Minister and the official AIDS policy was moved to this office. An intensive public information campaign on HIV/AIDS prevention was launched through mass media; all ministries provided education for the adoption of universal coverage and protection of vulnerable groups among their constituent populations in 2002. Thailand successfully met the 90-90-90 targets, with 89% of people living with HIV having a known HIV status, 72% receiving ART and 82% undergoing viral load testing (with 99% achieving viral suppression), thus preventing 5.7 million infections since 1991
Uganda	The first country to have a multisectoral AIDS HLC under the auspices of the President. This, with the active engagement of civil society and early emphasis on voluntary counselling and testing had satisfactory results, allowed to reach the 90-90-90 targets by 2020

HLC = High-Level Commission; ART = antiretroviral therapy.

## Malaria experience

Engagement at the highest political level was crucial in Africa, where the vast majority of malaria cases and deaths occur. A remarkable example was the campaign launched in 2018 by the President of Rwanda as Chair of the African Union ‘*Zero Malaria Starts with Me campaign’*. This was endorsed by African leaders at the 31^st^ African Union Summit with 14 African countries. Heads of state of various countries established councils to end malaria, featuring ministers, civil society and corporations.^14^ Finally, the African Leaders Malaria Alliance (ALMA), is a coalition of African Union Heads of State and Government, established to drive accountability and action against malaria, neglected tropical diseases (NTDs), reproductive, maternal, neonatal, child and adolescent health (RMNCAH) and nutrition.

## COVID-19 experience

Early on during the pandemic, the European Union (EU) recognised the ‘limits of its capacity to act decisively and exposed the lack of the Commission’s executive and budgetary powers’. This acknowledgement indirectly called for the involvement of the highest political authorities in the EU and its Member States.^15^ In most countries, it was the head of state who directed the multi-sectoral response to COVID-19. In Pakistan, a country with a highly decentralised health system and major coordination challenges, the National Coordinating Council (NCC) and National Command and Operation Centre (NCOC) were established to monitor the response to the pandemic. The NCC was headed by the Prime Minister and had representatives from all key ministries. NCOC served as the implementation arm of NCC. NCOC had provincial representation and relevant stakeholders, which included all major ministries. This coalition of public and private services, as well as of civilian and army forces at both the federal and provincial level, proved successful.^16^

## Are HLCs useful?

Assessing the impact of political commitment is difficult. Although one can try to identify benefits from high-level political leadership in terms of health outcomes, attributing success to a single action is often impossible. However, these limitations notwithstanding, HLCs have contributed to the 90-90-90 HIV targets in several countries, increasing the proportion of insecticide-treated mosquito nets and reducing malaria-related maternal mortality in Rwanda, and to responding to COVID-19 in many countries (including those with a decentralised system). The best examples come from the AIDS response, and examples in the field of TB are unfortunately limited. However, as mentioned above, the direct engagement of the head of state (in countries like Vietnam, India, Indonesia and Timor-Leste) suggests that progress may be accelerated when political commitment is at the highest level. One theoretical risk of HLCs reporting to the head of state is that of alienating the Ministry of Health and of creating uncertainty about accountability when too many authorities are involved and leadership is diluted. Lessons from the past indicated that it is imperative for the Ministry of Health to retain a prominent role with the backing of the head of state, while also supporting cross-sectoral political means to advance the fight against TB.

In conclusion, establishing high-level, multisectoral bodies that report to the head of the state is a necessary step to achieving high political visibility, and mobilising resources in all sectors. Without an elevated response that goes beyond the health paradigm to address social and economic aspects, the world will not be able to end TB. Additionally, this level of engagement of all relevant sectors may also help to alleviate the risk factors and determinants that are common to many other diseases of poverty. In this way, a focus on TB may act as a driver for transformational change.

## References

[1] World Health Organization. Sixty-Seventh World Health Assembly. Geneva, Switzerland: WHO, 2014.

[2] World Health Organization. First WHO Global Ministerial Conference on Ending TB in the sustainable development era: a multisectoral response. Moscow Declaration on Ending Tuberculosis, 17 November 2017. Geneva, Switzerland: WHO, 2017.

[3] United Nations. Political declaration of The UN General Assembly High level meeting on the fight against tuberculosis. New York, NY, USA: UN, 2018.

[4] United Nations. The 17 Goals - Sustainable Development - the United Nations. New York, NY, USA: UN, 2015.

[5] United Nations. UN General Assembly High-level Meeting on the fight against tuberculosis. Political declaration. New York, NY, USA: UN, 2023.

[6] World Health Organization. WHO South-East Asia Region commits to further enhance efforts to end TB, adopt Gandhinagar Declaration. New Delhi, India: WHO, 2023.

[7] World Health Organization. Global tuberculosis report, 2023. Geneva, Switzerland: WHO, 2023.

[8] World Health Organization. Multisectoral accountability framework to accelerate progress to end tuberculosis by 2030. Geneva, Switzerland: WHO, 2019.

[9] Delhi End TB Summit 2018. We in India are working towards eliminating TB by 2025: PM Modi, 13 March 2018. New Delhi, India. https://www.narendramodi.in/text-of-pm-s-address-at-the-inaugural-session-of-end-tb-summit-539297. Accessed November 2023.

[10] World Health Organization South East Asia. Delhi TB Summit: WHO South-East Asia countries commit to intensified efforts, concrete progress to End TB. New Delhi, India. https://www.who.int/southeastasia/news/detail/13-03-2018-delhi-tb-summit-who-south-east-asia-countries-commit-to-intensified-efforts-concrete-progress-to-end-tb

[11] Vietnam Prime Minister Official Letter No. 4791/VPCP-KGVX on the establishment of the National Commission on Ending Tuberculosis. 2019.

[12] Vietnam Plus. Available at: https://en.vietnamplus.vn/vietnam-sets-up-national-committee-to-wipe-out-tb/153845.vnp.

[13] UNAIDS. UNAIDS: the first 10 years, 1996–2006. Geneva, Switzerland: UNAIDS, 2008.

[14] Sokunbi TO, . Nigeria End Malaria Council: What to expect. Ann Med Surg 2022;82:104690. 10.1016/j.amsu.2022.104690 PMC9486447 36148088

[15] European Parliament. Joint Motion for a Resolution on EU coordinated action to combat the COVID-19 pandemic and its consequences. Strasbourg, France: European Parliament, 2020.

[16] Rizwan A, Naveed S, Salman Y. An analysis of policies, challenges and outcomes in Pakistan through co-creation of COVID-19 responses. Public Admin Policy 2023;26(1):107–119.

